# Technics of Animal Vaccination*Read before the Pennsylvania State Sanitary Convention at Philadelphia, May 15, 1886.

**Published:** 1887-07

**Authors:** W. L. Zuill

**Affiliations:** Professor of Obstetrics, Surgery and Clinical Surgery, Veterinary Department, University of Pennsylvania


### ART. XXIII.—TECHNICS OF ANIMAL VACCINATION.*

BY W. L. ZUILL, M. D., D. V. S.

*Professor of Obstetrics, Surgery and Clinical Surgery, Veterinary Department, University of Pennsylvania.*

Since the discovery of vaccine virus, and proof that it gave immunity to Small Pox, one great question has vexed the world of medical science. It has asked how can we obtain an absolutely pure virus, the use of which will not be attended with the danger of inoculating the patient with some specific disease. This danger, while very great, in the use of humanized lymph, is very much diminished when bovine lymph is substituted.

This question of obtaining pure bovine lymph has reached its highest development in the countries of Continental Europe where the production of this material is under the supervision of the different governments, who have placed it in the hands of qualified veterinarians, thus obtaining the best guarantee possible for its purity. Does the same qualification hold good in this country? I am sorry to say that it does not. How many of the hundred so-called Vaccine farms are under the guidance of men who are qualified by reason of their training and education to distinguish the difference between bovine tuberculous and contagious pleuro-pneumonia, or to recognize this latter disease from that of a sporadic character.

I assert that any who are willing to take the trouble will find that a very small proportion of these farms are under scientific guidance of any kind, even the most questionable.

Vaccine, whether it be obtained from the equine or bovine race of animals, possess the same characteristics and if inoculated into the human system confers immunity from small pox, or a second vaccine inoculation. It has been considered that the characteristic properties of Vaccine was inherent in

---

*Read before the Pennsylvania State Sanitary Convention at Philadelphia, May 15, 1886.

the liquid. This is a mistake. It has been demonstrated beyond the possibility of a doubt that the active principle of vaccine is due to the presence of small special bodies which by every one is now admitted to be infectious parasitic vegetable organism. These microbes considered as the specific agents of small pox and vaccine are small perfectly spherical micrococci. Chauveax has undeniably demonstrated that vaccine lymph deprived of these corpuscular elements loses all its physical characteristics. While the microbe of vaccine does not differ in its visible characters from the small pox microbe, I do not desire to be understood as saying that vaccine is small pox attenuated by its passage through the body of the horse or cow. Kelb mentions as a characteristic which is more or less common to the microbe of vaccine as to those of small pox the disposition to form groups of four, and has therefore given them the name micrococcus quadrigeminus.

For nearly one hundred years the desire to determine the question whether the vaccine which we employ to protect ourselves from small pox came in the first place from the horse or cow, has given rise to the most animated discussion in the medical world. Jenner in his own work published in London in 1798 entitled, "An inquiry into the cause and effects of Variola Vaccina", admits that vaccine was originally derived from the horse, recognizing that certain persons employed to care for milch cattle were not susceptible to the small pox virus with which he inoculated them, and that they owed this immunity to a disease which they had contracted from the udders of the animals which they were in the habit of milking, but at this point he has been obliged to admit that these cattle had taken the disease from horses with which they were directly or indirectly in contact. This disease of the horse is spoken of by English writers of veterinary literature as grease; this misapplied cognomen is likely to lead one astray and to divert our attention from the true constitutional disease, termed by M. Bouley Horse pox, to that purely local one called as I have said by English writers—grease. Cow pox, may be produced in the cow by inoculating with horse pox, with natural or artificial cow pox or by human vaccine ; upon this property depends the practice known as *animal vaccination*.

The vesicle of vaccination in the horse presents different characters from those which are found in the cow or in man. In the horse they present a conical appearance, instead of being umbilicated, as is found in the others. The secretion of the vesicle is also different in the horse ; that from the entire vesicle is effective ; while in the cow and man that from the centre of the vesicle only presents any activity. What is this special contagion ? Is this contagion the same for man as for the horse or cow, or are there different contagions for each? The solution of these questions depend upon the similarity of the germ of small pox and of vaccina. They have for more than half a century been the subject of the most interesting discussions, and to-day they may be said to be altogether unchanged and undecided. It remains for modern scientists to eclucidate the facts from these questions.

*The technics of animal vaccination.* Up to the present time vaccination termed animal has been resorted to only in the bovine species. I prefer to use for this purpose small animals varying from six to eighteen months of age ; although an advanced age is no more serious objection than what would naturally follow from an increase in size. These calves should weigh from two to five hundred pounds and be in good health. It is seldom I can obtain animals that are fit for immediate vaccination ; as a rule they come to us in a very unthrifty condition. They have been wintered in the barn-yard, often without even a shed for shelter, their food which at the best has been corn stalks, has left them in such condition that unless much care is exercised in the first ten or fourteen days the change of food will be sufficient to bring on an attack of diarrhœa. Notwithstanding the great care exercised in the change of food of these animals, it is by no means uncommon to see them suffer from slight attacks of this disease. The first thing necessary after the animal has reached our hands is to rid its hide of the lice and other vermin which infest it, this is absolutely essential for obvious reasons ; while the animal is running in the field or yard it appears to suffer little or no inconvenience from these insects which are benumbed with cold, but immediately they are put into a warm stable they will begin to itch and consequently rub, bite and scratch themselves, their temperature will go up to 103° or more, the hair will begin to fall, and in a short time they are sorry looking objects. In order to rid the animals of the vermin it is only necessary to give them two or three baths of sheep dip ; this, with a week or ten day's careful feeding, is all that is required to bring the animals into condition to be vaccinated. Color is a question of importance in the selection of animals; a red and white coat indicate a soft skin, one that is free from black pigmentation is an advantage in our observation of the different stages of the eruptive fever. When the animal is in proper condition, it is brought into the operating room where it undergoes a most rigid examination. There must not be any diarrhœa or digestive derangement of any kind, neither must there be any bruise, wound or abrasion, broken horns or hoofs of recent date, as complications of this kind will prevent the take of the vaccination. Animals upon which are found neoplasms of any kind are also rejected. Elevation of temperature, increased respiration and pulse with other symptoms of acute disease are carefully looked for and as carefully rejected when found. An exhaustive and thorough examination is made to determine the presence of lung lesions, and the slightest indication of their presence is sufficient to prohibit the utilization of that animal for the propagation of vaccine lymph. The animals which are to be used for vaccinating should be confined in large roomy stables. Large windows should furnish light to this building, ventilation and drainage to be as perfect as it can be made. The food should consist of bran, ground corn, oats and cut hay supplied in liberal quantities. Straw for bedding must be used freely. A bed twelve to fourteen inches thick to be kept under the animals constantly ; they should be carefully groomed twice every day, and as much pains taken with their coats as if they were race horses.

When it is desired to vaccinate an animal it is led to the operating-room, and there placed upon an upholstered table, upon its back, its legs upward, each of which after being protected by a pad of thick felt is securely fastened to a post in the frame of the table ; while in this position the hair is removed from the inside of the thighs and the belly, as far forward as the umbilicus. The hair is first removed with the scissors and soaped and shaved perfectly clean ; upon the shaved surface are afterwards placed from fifty to seventy or even one hundred points of insertion, varying in size from a quarter to a half dollar. These points are made by first scraping off the epidermis and afterwards making a number of scarifications in different directions.

Then comes the important step of the operation, placing on the vaccine ;—This should be done with the greatest care as upon this depends success; it is truly remarkable what an astonishing number of failures can be obtained with the best virus, when the operation is not properly performed. The animals, after being vaccinated are placed in a warm stable, and where there will be no danger of drafts or cold to which they are now very susceptible. The stalls in which they are placed must be wide enough to be comfortable, but sufficiently narrow to prevent the animal from turning, which it will invariably try to do in order to lick the scarifications which burn and sting very much. Abundance of clean straw must be supplied and the fæces from the animals removed as fast as they fall. This insures cleanliness which is one the great essentials of success.

We must not forget that the full product of vaccination should be obtained on the sixth day at the farthest, and in Europe when all the animals are slaughtered for food they are always sent to the butcher on the seventh day, or before the posible development of suppurative fever.

It is a fact that the animals do not suffer through vaccination, how numerous so ever the insertions may be ; it may suffer from the effects of a journey, or from ill treatment it has received, from change of stable, or companions but from vaccination never, nor is vaccination ever a cause for depreciation in its value.

It is an unanswered question why at or during the vaccinal period calves are likely to be attacked with diarrhœa and tympanitis? Diarrhœa will often yield to light and proper diet and some emolient drinks in a few hours; if it persists, it may arrest or at least delay vesication. Tympanitis, which is a frequent complication is a disagreeable one; if this complication should arise at the beginning of the eruption, it modifies it at once. The vesicles become flattened and may even disappear or dry up completely. Should the vesicles be full when the stomach trouble comes on, the flow of lymph will decrease and in a few moments entirely disappear. All that can now be done is to treat the tympanitis and wait results. A few hours often is all that is necessary for the skin to regain its softness and for the vesicles to return to their normal condition.

Notwithstanding the great care exercised to prevent the vesicles on the belly of a heifer from being torn or broken they will be more or less injured; this is an accident difficult to avoid, and one of little importance.

If the surface of the vesicles are torn they need not on this account lose either their contents or their intrinsic value; for this reason there is no necessity to try to avoid this accident of tearing, as could be done by placing a pad over the abdomen of the animal and by changing the ordinary litter, for a floor with an opening, which would allow the escape of excrementitious matter. The greatest danger to the vesicles, and one which must be carefully guarded against, is the animals licking the wounds and thus wash away the vaccine just deposited there; this itching sensation which causes the animals to lick themselves is evident immediately after the vaccination and during the irruptive stage.

We can avoid this means of sterilization and bruising by placing the animal in a stable in which it cannot turn, or by putting on a muzzle; for the same reason it is always advisable to secure the tail of the animal, which can be easily done by placing a splint upon it; thus preventing it from being bent. This simple procedure to a great ex-tent lessens the ease with which the animal approaches its mouth to the vaccinated region.

The collection of the vaccine lymph is a procedure which requires a great deal of care and judgment or in all probability you will collect nothing but inactive serum.

Vaccine lymph is nothing but serum holding in suspension small spherical bodies micrococci, which constitutes the active principle of the lymph. The presence of these bodies gives to the lymph a thick mucilaginous character; this gummy feel is altogether wanting in serum which has not the active principle of vaccine. The lymph may be collected from the vesicles under pressure of forceps made for this purpose and is not by any means an objectionable feature. After the scab and other foreign matter has been removed from the vesicle they should be gently pressed with a clean linen rag slightly damp, and should blood flow, it must be removed in this way until it has entirely stopped. In a few moments serum or lymph will start which may be recognized by the following test: take a small quantity between the fingers and thumb, if it is sticky as a solution of gum, it is *prima facia* evidence that the lymph is good, but if this sensation or condition is not to be had, then the vesicle must be abandoned. If it is desired to collect the vaccine lymph in tubes, it is allowed to form on the surface of the vesicle in little pools, and in the pools thus formed we may place a vacuum tube or a capillary tube open at both ends which are soon full; such tubes, however, are of little value as they become useless in a few hours. When mixed with equal parts of distilled water and glycerine it may keep five or six days.

*Ivory Points.*—This is certainly the best method of preserving animal vaccine in a dry state; these points when properly prepared will retain their activity for three or four weeks. In every instance the points should be double charged; the second charge must not be made from the animal which supplied the first; this gives greater security, for should the first charge have been made from a negative animal or from a negative vesicle only by the slightest chance would the second charge be of the same character. Points destined for a long voyage are tripled charged, and afterwards dipped in a solution of gum arabic and the whole covered with tin foil.

*Vaccine in pulp.* — The vesicle alone. This barbarous practice was used in the earlier days of animal vaccination, it consisted in excising the vesicle with a portion of the skin down to the subcutaneous connective tissue. As far as the results of this method are concerned, I have nothing to say ;—as, with an execution it is merely the brutality of the practice—the wound made on the abdomen of the animal from which blood flowed in streams, and the uncanny appearance of the piece of skin, scarcely an attractive object to place before the person to be vaccinated, but in order that the results of this practice be satisfactory, it must be used at once.

*Glycerinized Pulp.*—This method consists of using the whole vesicle ; it was first introduced in Milan when they excised the skin with the vesicles which they fix to a board with strong pins. The vesicles are scraped and with this scraped skin they make a glycerinized pulp or paste; this is placed in a bottle and a little pure glycerine placed on it which acts as a cork or stopper to exclude the air. This pulp was improved on by Warlomont by removing from the surface every impurity, even the so-called vaccine scab. After this is done he says that by a special process the core of the vesicle is reduced to a thin mass, this is treated with glycerinized water and the emulsion obtained is put in cylindrical tubes of amber colored glass. This emulsion keeps so well that he advises vaccinators to constantly carry one of these phials in their cases for use at times of need.

Doubtless this emulsified animal tissue—and this is what it must be if it is the scraped vesicle—may be good enough for European practitioners, but I would not ask an American practitioner to use any such compound. I believe that I have made a glycerinized vaccine which is very much better than Warlomont's, even as much superior as his is an advance on the old method of using the entire vesicle. The process is remarkably simple.

The first step is to thoroughly cleanse the vesicle of every particle of foreign matter and as the pure clean lymph wells up from the depths of the vesicle to collect it in a small spoon —it is possible not to collect shreds of tissue and foreign matter at the same time—the lymph as collected is placed in a small phial and an equal weight of chemically pure glycerine is incorporated with it, this is afterwards filtered not only making a beautifully clear liquid but also removing all foreign matter.

This solution of glycerine of lymph, will keep, and be absolutely sure in its results for a number of months.

